# Assembly and priming of the VPS4B AAA+ ATPase of the human ESCRT system

**DOI:** 10.21203/rs.3.rs-9696998/v1

**Published:** 2026-07-31

**Authors:** James Hurley, Yuchao Zhang, Shuixia Tan, Kevin Larsen, Sumin Kim, Lilah Byun, Isabella Alfonso

**Affiliations:** University of California, Berkeley; UC Berkeley; UC Berkeley; UC Berkeley; UC Berkeley; UC Berkeley; UC Berkeley

## Abstract

Vacuolar protein sorting-associated protein 4 (VPS4), which occurs in A and B isoforms in humans, is the only enzyme in the core endosomal sorting complex required for transport (ESCRT) machinery. Human VPS4 is considered a potential therapeutic target for activation in neurodegeneration, and for inhibition in cancer and HIV-1 infection. VPS4 assembles transiently into a hexamer, which then removes ESCRT-III subunits from polymeric assemblies by unfolding them and threading them through its central pore. The N-terminal MIT domain of VPS4 binds to C-terminal MIM motifs of ESCRT-III. Here, we determined the cryo-electron microscopy structure of the full-length human VPS4B hexamer in six-membered helical “spiral staircase” states. In one of these states, two of the six MIT domains are ordered and stabilize the hexamer by bridging the seam of the spiral staircase. Residues involved in seam-bridging contacts were found to be important for biochemical and cellular activity. The structures also revealed two modes for polypeptide occupancy of the central pore, one of which involves the MIT-AAA linker peptide. These observations suggest a mechanism for substrate-dependent hexamerization and priming of VPS4 for its ESCRT-III remodeling activity.

## Introduction

The endosomal sorting complex required for transport (ESCRT) machinery was discovered and named for its role in internalizing integral membrane proteins into the lumen of endosomes in the endolysosomal pathway in yeast^1^. Shortly thereafter, the ESCRTs were shown to mediate the release of HIV-1 particles from infected human cells^2–5^. In the wake of these discoveries of such divergent roles from yeast to humans, subsequent work in many labs made clear that “ESCRTs are everywhere”, crucially involved in a vast array of cellular processes ranging from cytokinesis to membrane repair and organellar quality control^6,7^. A common theme in ESCRT mechanism is “reverse-topology” membrane scission, the severing of membrane buds and necks that are directed away from the cytosol. These processes include multivesicular-body biogenesis^8^, cytokinesis^9^, nuclear envelope reformation^10,11^, autophagosome closure^12^, plasma and lysosomal membrane repair^13–15^, in addition to HIV budding^16,17^.

The core ESCRT machinery consists of ALIX, ESCRT-I, ESCRT-II, ESCRT-III and the AAA^+^ ATPase VPS4^18,19^. The upstream components ALIX, ESCRT-I and -II are mainly involved in cargo recognition and budding initiation. ESCRT-III consists of seven proteins in yeast and twelve (CHMP1A/B, CHMP2A/B, CHMP3, CHMP4A/B/C, CHMP5, CHMP6, CHMP7, and IST1) in humans. ESCRT-III proteins are inactive as cytosolic monomers and function by forming spiral^20^ and helical^20–22^ assemblies of loosely-defined stoichiometry at membrane necks. VPS4, a member of the meiotic clade of type I AAA^+^ ATPases^23^ is the sole enzyme in the core ESCRT machinery^24^. VPS4 is responsible for remodeling the ESCRT-III assembly to drive scission^25,26^, and, subsequently, the disassembly of the ESCRT-III complex and the recycling of its subunits to the cytosol.

Most of what we know about the mechanism of ESCRT-III remodeling by VPS4 comes from studies on the ortholog from the budding yeast *Saccharomyces cerevisiae* (hereafter, “yeast”). Vps4 functions as a hexamer^27^, like many other AAA^+^ ATPases. The assembly of Vps4 into hexameric rings is transient, which has posed a persistent challenge to its structural analysis. The cryo-EM structure of yeast Vps4 has been determined as stabilized by ADP·BeF_x_ and fusion to the hexameric scaffolding partner Hcp1^28^, or by the catalytic mutation E233Q^29,30^. Vps4 unfolds ESCRT-III proteins by threading them through the central pore of the hexamer as shown by HDX-MS^31^ and cryo-EM^32^. The pore can accommodate two polypeptide chains at once^33^. The most extensive series of analyses have been carried out using the Vps4-Hcp1 construct. These structures show that five of the six subunits have helical symmetry. The sixth subunit occupies a seemingly transitional position intermediate between the start and the end of a turn of the helix. The data collectively are consistent with a hand-over-hand model in which ATP hydrolysis perpetuates cycles of helix assembly at the leading end and disassembly at the lagging ending, thereby threading the polypeptide substrate through the pore^33^.

VPS4 initially engages with its substrates through its N-terminal microtubule interacting and transport (MIT) domain^34,35^. MIT domains are three-helix bundles that bind short MIT-interacting motifs (MIMs) located at the C-termini of ESCRT-III proteins. MIMs exist in multiple subtypes, of which two predominate among the ESCRT-III proteins, the a-helical MIM1 of most CHMPs and the extended MIM2 of CHMP6^36^. The avidity of VPS4 recruitment to polymerized ESCRT-III is thought to be driven by the presence of six MIT domains per hexamer. The VPS4 activator VTA1 (also known as LIP5) also contains ESCRT-III-binding MIT domains^37–39^. VTA1 dimerizes and binds to VPS4 via its Vta1/SBP1/LIP5 (VSL) domain^40–42^. The VSL dimer bridges two adjoining AAA+ domains in the hexamer^28,30^, thereby promoting hexamerization and ATPase activity. ESCRT-III proteins are themselves autoinhibited by their C-terminal regions^43^. Their MIMs are exposed following polymerization on the membrane. Engagement with VPS4 MIT domains in turn promotes the activation of the VPS4 ATPase^39^. In the yeast system, it has been proposed that the MIT domain can sterically block access to the central pore and so block VPS4 ATPase activity in the absence of ESCRT-III engagement^44,45^. This implies a physical interaction between the MIT domains and the ATPase ring, however, this has not been directly observed in an experimental structure determination. Thus, communication between the MIT domains and ATPase core is central to understanding VPS4 regulation, yet has never been elucidated structurally.

An intronic deletion gene encoding the ESCRT-III protein CHMP2B that removes the MIM is responsible for familial frontotemporal degeneration^46^. This, and the broad importance of the ESCRT system in quality control suggests potential for therapeutic activation of VPS4 in neurodegeneration^7^. VPS4A and VPS4B are often chromosomally co-deleted with tumor suppressor genes in colorectal, pancreatic, and other human cancers^47,48^. The requirement for VPS4 function for the viability of dividing cells suggests isozyme-specific inhibition of human VPS4A/B as a potential therapeutic strategy in cancer^47,48^. Targeting the ESCRT system in HIV-1 and other viral infections is also thought to have potential^49^. The targeting of VPS4A/B for either activation or inhibition should be greatly facilitated by a better understanding of its physiological regulatory mechanisms and structure. Yet the only structures available for human VPS4 are a crystal structure of the monomeric ATPase domain of VPS4B^42,50^ and structures of isolated MIT domains^36,51,52^. As interest in pharmaceutical targeting of human VPS4 increases, it is becoming increasingly urgent for the field to have a complete structural picture of at least one, if not both, of the human VPS4 isoforms.

## Results

### Cryo-EM structure of human VPS4B^E235Q^-Hcp1 hexamer in a transitional state

**Figure 1:**
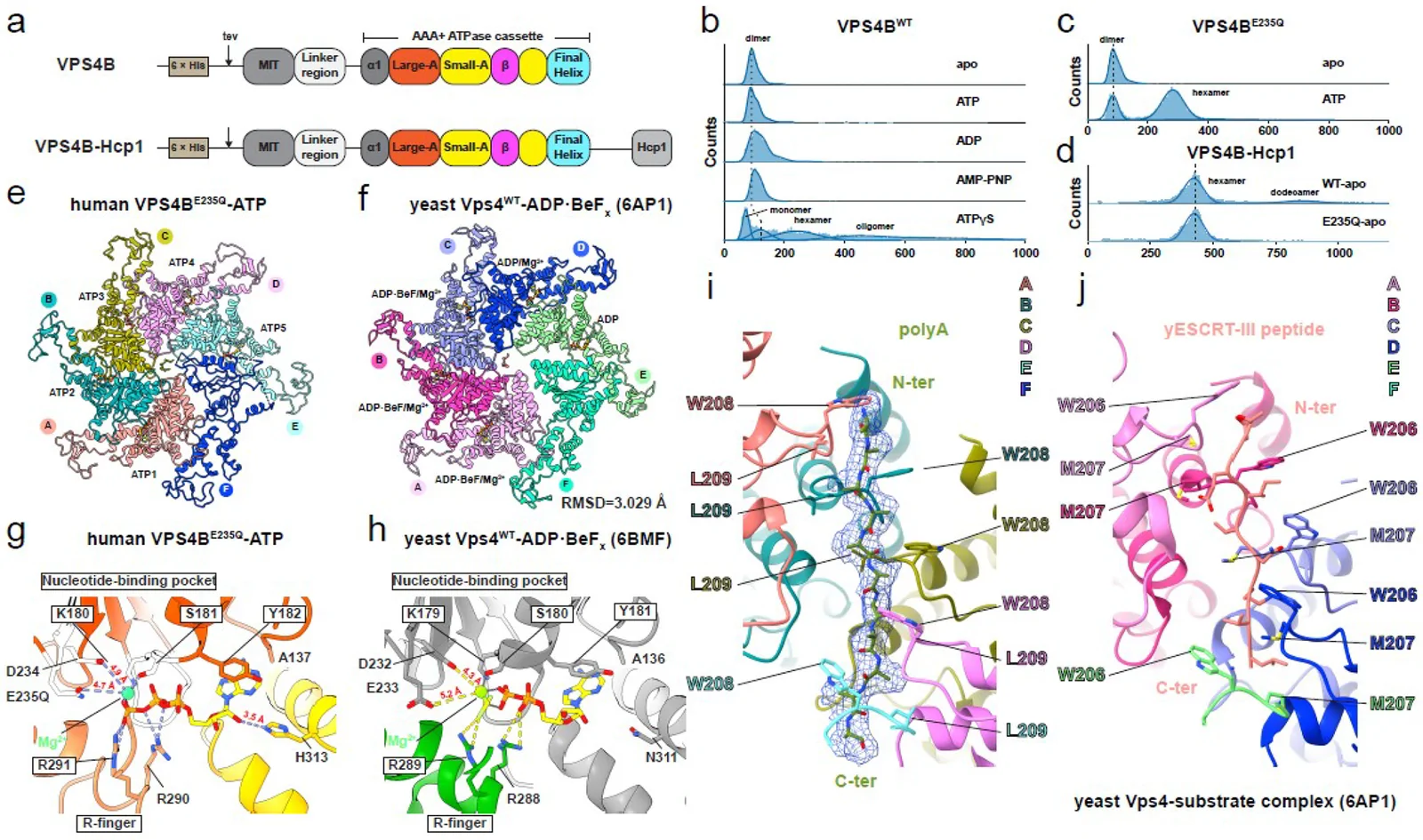
Architecture of human VPS4B^E235Q^ hexamer. **a**, Construction and domain organization of human VPS4B. α1 indicates the first α-helix of ATPase cassette; β, β domain. **b-d**, Mass photometry measurements of human wild-type VPS4B in the presence of different ATP analogs. As indicates, ATP, ADP and AMP-PNP cannot promote human wild-type VPS4B to form hexamer. Only ATPγS can promote human VPS4B hexamer or oligomer formation (**b**). Human VPS4B dominant negative form (E235Q) in the presence or absence of ATP (**c**). Measurements of both wild-type and E235Q mutant using C-terminal Hcp1 fusion constructs (**d**). **e**, Overall structure of human VPS4B^E235Q^ hexamer in top view. **f**, Overall structure of yeast wild-type Vps4 hexamer (PDB: 6AP1)^32^ in top view, and the root mean square deviation (r.m.s.d.) value between human VPS4B^E235Q^ hexamer and yeast wild-type Vps4 hexamer is ~3.029 Å. **g-h**, Close-up views of human VPS4B^E235Q^ (**g**) and yeast wild-type Vps4 (PDB: 6BMF)^32^ (**h**) nucleotide-binding pocket. The related hydrogen bonds involved in the binding are shown as dotted lines. **i**, Close-up view of VPS4B hexamer central pore. Density map shows the pore peptide. Residues Trp208 and Leu209 of subunits A-E are mainly responsible for packing against the pore peptide. **j**, Close-up view of yeast Vps4 hexamer central pore bound with yeast ESCRT-III substrate (PDB: 6AP1). Residues Trp206 and Met207 of subunits A-E are mainly responsible for packing against the yeast ESCRT-III substrate.

Human VPS4A and B are both composed of an N-terminal MIT domain, followed by a MIT-AAA^+^ linker region and an AAA^+^ ATPase domain. The AAA^+^ ATPase domain can be further divided into large and small ATPase domains and a unique β domain, all bookmarked by N- and C-terminal a-helices^42^ (Fig. 1a). The transient nature of VPS4 hexamerization presents a major challenge to its structural characterization. Using mass photometry, we found that wild-type human VPS4B predominantly exists as dimers in solution in the absence of nucleotide or in the presence of ADP, ATP, or the non-hydrolyzable ATP analog AMPPNP (Fig. 1b). In the presence of the slowly hydrolyzable analog ATPgS, VPS4B is predominantly monomeric, with trace amounts of dimer, hexamer, and higher oligomers present (Fig. 1b). The catalytically inactive mutant VPS4B^E235Q^ exists as dimers in the absence of nucleotide, and as a mixture of dimer and hexamer in the presence of ATP (Fig. 1c).

The cryo-EM structure of hexameric wild-type Vps4 from yeast was solved by using a C-terminal fusion to the hexameric bacterial protein Hcp1^28,32,33,53^. Hcp1 has no biological relationship to the ESCRT system and was used solely to promote hexamerization. To maximize the comparability with the yeast work and generate a maximally monodisperse population of VPS4B hexamers, we generated a human VPS4B-Hcp1 construct (Fig. 1a). Mass photometry showed that the wild-type VPS4B-Hcp1 fusion construct is predominantly hexameric, even without nucleotide (Fig. 1d). However, the VPS4B portion of the fusion was found by cryo-EM not to assemble into a hexamer, even in the presence of ATP. Therefore, we generated a VPS4B^E235Q^-Hcp1 construct, and found the VPS4B portion to be hexameric in the absence of ATP (Fig. 1d) and to form a well-ordered structure tractable to cryo-EM structure determination (Fig. 1e). The final map was reconstructed by subtracting the Hcp1 density, and the overall resolution is 2.90 Å (Extended Data Fig. 1a-l). Five chains of the six in the hexamer are well-resolved, while for the sixth (denoted the F chain), the local resolution of subunit is ~6 Å (Extended Data Fig. 1m).

The human VPS4B^E235Q^-Hcp1 hexamer bound to ATP has a qualitatively very similar overall organization to the yeast wild-type Vps4 hexamer bound to ADP·BeF_x_. The Ca root mean square deviation (r.m.s.d.) value of two structures is 3.0 Å when the entire hexamer is overlaid, and 1.2 Å on a monomer basis for the well-ordered A-E subunits. In both cases, subunits A-E conform to a regular right-handed helical pattern. The F subunit occupies a position transitional between the top and bottom of the helix, as though caught in the act of translocating from one position to the other. For this reason, we refer to this state of the hexamer as “transitional”. There are some subtle differences in that subunit F in the human VPS4B^E235Q^ hexamer is closer by ~10 Å to the central pore than in yeast Vps4^WT^ hexamer (Fig. 1e–f). Within the nucleotide-binding pockets, small differences are seen that we attribute to the use of the E235Q mutation in the human structure, one naturally occurring amino acid substitution in the catalytic site of the human *vs*. yeast enzymes, and the presence of different nucleotides (Fig. 1g–h)^32^. Beyond the highly conserved nucleotide binding site, the nature of the subunit interfaces is well-preserved (Extended Data Figs. 2 and 9a). This suggests that the use of the EQ mutation to stabilize the human structure as compared to ADP·BeF_x_ for yeast did not introduce material differences. The similarity lends plausibility to the idea that the mechanism of ATP-drive ESCRT-III disassembly is essentially conserved from yeast to humans.

Focusing on the hexamer central pore, we observed clear density for a single peptide chain (Fig. 1i), whose orientation resembles that of yeast Vps4 (Fig. 1j). The density for the peptide was insufficient to build side chains, therefore it was modeled as polyalanine. The pore loop residues Trp208 and Leu209 of each subunit form a hydrophobic channel for substrate threading (Fig. 1i).

### Cryo-EM structure of human VPS4B^E235Q^ hexamer in the processing state

A major motivation for this study was to map the complete conformational pathway for regulation, initiation, and processing of substrate by VPS4B, with the ultimate goal of facilitating activator and inhibitor development. We speculated that it should be possible to visualize all six subunits in a well-ordered regular helix. Moreover, given evidence for MIT domain-ATPase core interactions^44,45^, we hypothesized that is should also be possible to visualize the interactions of at least some of the MIT domains with the core. We hypothesized that the presence of the Hcp1 fusion might bias the equilibrium between states, potentially leading to the depopulation of relevant states from the observed ensemble. We therefore sought to determine unfused VPS4B hexamer structures.

Human VPS4 is activated by the ancillary MIT domain-containing protein LIP5/VTA1^39,42^. Gel filtration results show that VPS4B^E235Q^ and VTA1 could form a complex in the presence of ATP^54^ (Extended Data Fig. 3). To further stabilize VPS4B^E235Q^ hexamer core in the absence of Hcp1, we expressed and purified VTA1 and VPS4B^E235Q^ separately, mixed them, and co-purified the VPS4B^E235Q^/VTA1 complex by size-exclusion chromatography (Extended Data Fig. 4a). Image processing and 3D reconstruction resulted in a cryo-EM reconstruction in two classes. The more populated class had an overall resolution of 2.69 Å, and the less populated class, 3.32 Å (Extended Data Fig. 4b-d). Although we used VPS4B^E235Q^-VTA1 complex for data collection, we could not observe any VTA1 density in either class, possibly because of interference with the stability of the VTA1 complex from the air-water interface or the grid material.

**Figure 2:**
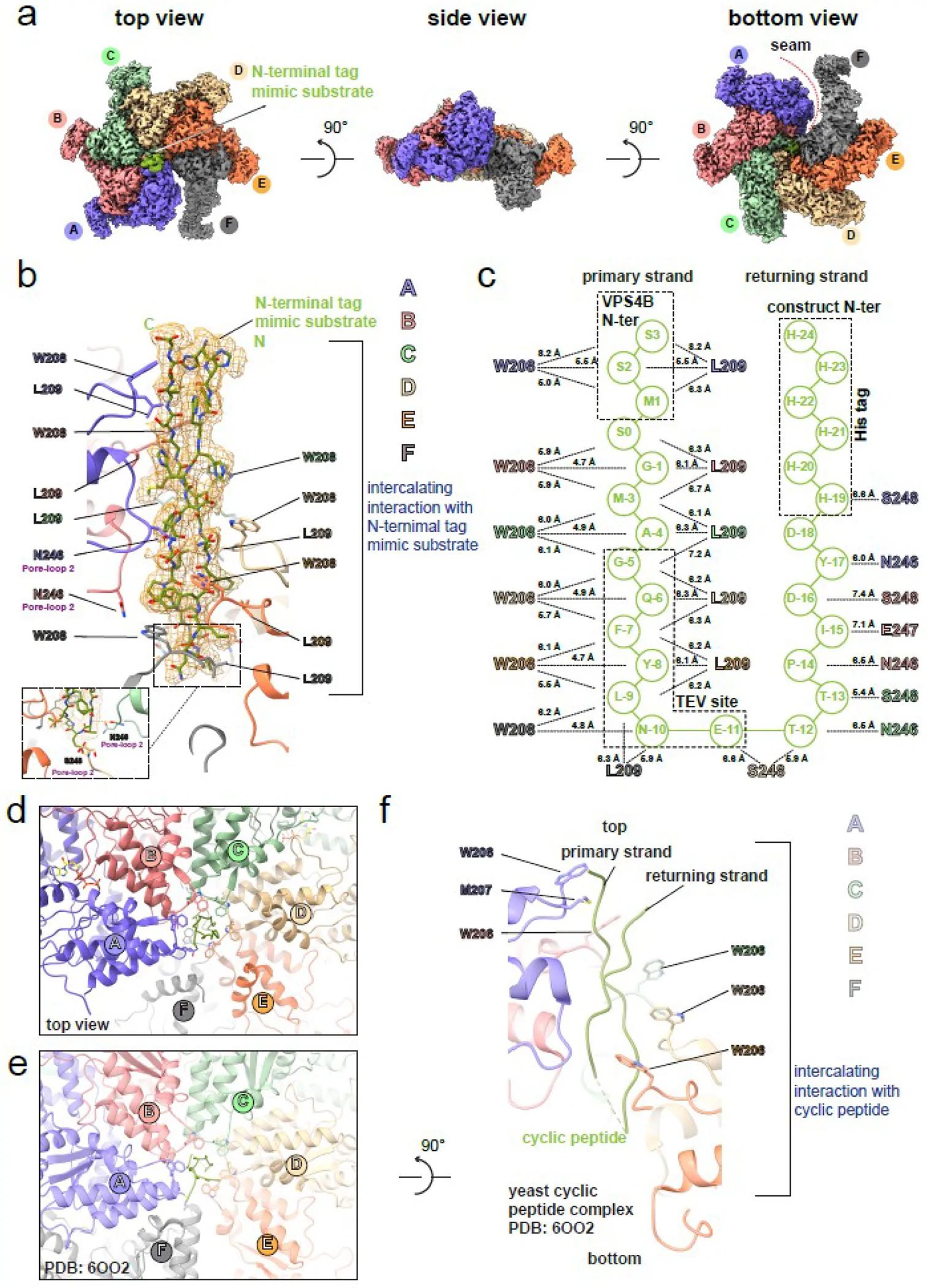
Human VPS4B^E235Q^ hexamer in processing state. **a**, Cryo-EM map of human VPS4B^E235Q^ hexamer in processing state. **b**, Close-up view of VPS4B processing state central pore. Density map of N-terminal tag mimic substrate is clearly to build residues. Zoom-in panel shows the hairpin turn feature in the central pore and related interaction residues. **c**, Cartoon representation shows the distances between Cα atoms of the N-terminal tag mimic substrate and pore loop 1 Trp208 and Lue209. The subunits A-D pore loop 2 residues involving in the interaction with N-terminal tag mimic substrate are also indicated. **d-e**, Top view of processing state central pore (**d**) and yeast Vps4 hexamer central pore bound with cyclic peptide (PDB: 6OO2)^33^ (**e**). **f**, Close-up view of yeast Vps4 hexamer central pore bound with cyclic peptide. Residues Trp206 and Met207 of subunits A-E are mainly responsible for packing against the primary strand of cyclic peptide.

The hexameric structure obtained from the most populated class consists of six copies of VPS4B ATPase domain, with well-defined density for the N-terminal tag in the central pore which may mimic substrate binding. All six ATPase domains participate in a regular helical arrangement, with an exposed seam between subunits A and F (Fig. 2a). The nucleotide-binding pocket well-resolved for all six subunits, and the density clearly confirms that ATP and Mg^2+^ are bound to each subunit (Extended Data Fig. 4e and 7a). We were able to assign the residues bound in the pore, on the basis of the high resolution of the density in this region. The N-terminal tag is composed of His_6_ tag followed by an engineered linker, a TEV site, continuing into the first three residues of VPS4B, Met-Ser-Ser, which were all clearly resolved. The interaction is very extensive, burying a total of 1715 Å^2^ solvent accessible surface area. The pore loop residues Trp208 and Leu209 from all six subunits are in direct contact with the N-terminal sequence. Because the N-terminal sequence as seen in this class appears to mimic a substrate in the process of translocation, we will refer to this most populated class as the “processing” state.

The N-terminal tag forms a twisted b-hairpin, showing that the pore of human the VPS4B hexamer can simultaneously accommodate two peptide chains (Fig. 2b–c). The N-terminal b-strand mainly packs against the hydrophobic ladder formed by the hydrophobic residues Trp208 and Leu209 from pore loop 1, while the second strand is held against pore loop 2 mainly by peptide backbone contacts (Fig. 2b–c). The six ATPase domains form a spiral staircase that embraces the hairpin strands (Fig. 2d). Yeast Vps4 was previously shown to be able to bind a cyclic peptide in the central pore^33^, however, subunit F in the yeast hexamer is far from cyclic peptide (Fig. 2e). While the first strand engages similarly with pore loop 1 in the yeast cyclic peptide complex^32^, the second strand is only loosely bound in the yeast Vps4-Hcp1 structure (Fig. 2f). The first strand is aligned in the same direction as in the single peptide bound form of the yeast Vps4 hexamer^32^. Our interpretation is that normally all six subunits can simultaneously engage with substrate.

### Ordered MIT-AAA linker and MIT domains in the structure of VPS4B^E235Q^ hexamer primed state

**Figure 3:**
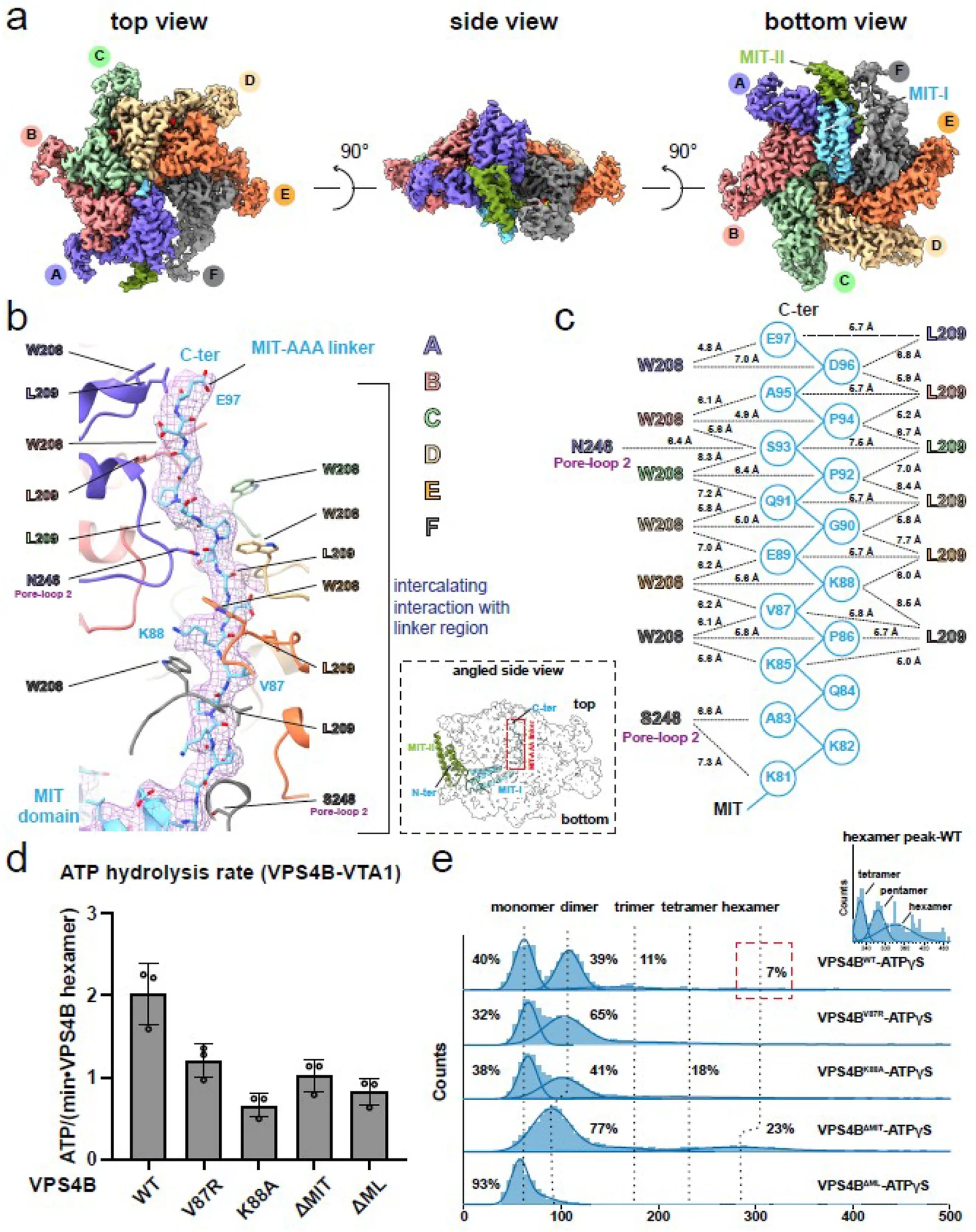
Human VPS4B^E235Q^ hexamer in primed state. **a**, Cryo-EM map of human VPS4B^E235Q^ hexamer in primed state. **b**, Close-up view of VPS4B primed state central pore. Density map of the MIT-AAA linker is clearly to build residues. The related hydrogen bonds involved in the binding are shown as dotted lines. Angled side view shows the MIT-AAA linker region C-terminus is out from hexamer top side, and MIT-I and MIT-II domain fill the seam between subunit A and F. **c**, Cartoon representation shows the distances between Cα atoms of the MIT-AAA linker and pore loop 1 Trp208 and Leu209. The pore loop 2 residues involving in the interaction with MIT-AAA linker are also indicated. **d**, ATP hydrolysis rate of human VPS4B and corresponding mutations or truncations in the presence of human VTA1. A total of 0.8 μM VTA1 was incubated with 0.4 μM VPS4B and 1 mM ATP for 30 min at room temperature (n = 3 independent experiments). **e**, Mass photometry measurements of VPS4B and corresponding mutations or truncations incubated with 1 mM ATPγS for 30 min at room temperature in the absence of human VTA1. Inset shows the zoom-in area of hexamer in wild-type profile. A total of 80 nM protein is used for measurements.

The less populated of the two 3D classes obtained in the absence of Hcp1 fusion also manifested six copies of ATPase domain in a regular helical arrangement, as well as two ordered copies of VPS4B MIT domain (referred to a MIT-I and -II) and central pore occupancy by a single polypeptide strand corresponding to one MIT-AAA^+^ linker (Fig. 3a). The MIT-I and -II domains bury 426 Å^2^ and 558 Å^2^ of solvent-accessible surface area, respectively, against the ATPase core (Fig. 3a). The two MIT domains bury a further 735 Å^2^ between them, suggesting a high degree of cooperativity in their mutual interaction. Indeed, no evidence was seen for 3D classes containing only single ordered MIT domains. Although the overall resolution of the primed state density is not as high as for the processing state, the local resolution of the nucleotide-binding pocket is excellent, and shows occupancy by ATP and Mg^2+^ in all six nucleotide pockets (Extended Data Fig. 4f and 7b), just as in the processing state. Therefore, the differences in the structures are not caused by differences in nucleotide binding site, but rather reflect different conformations that can exist simultaneously in the sample. MIT-I is located near the pore at the center of the seam, while MIT-II is clamped between subunits A and F towards the periphery of the seam (hereafter MIT-II) (Fig. 3a). As described below, we infer from structural reasoning that this mode of MIT domain docking is incompatible with substrate processing, and it more likely involved in priming the reaction. Therefore, we will refer to it as the “primed” state.

The MIT-AAA^**+**^ linker residues are clearly defined in the density, and can be modeled continuously from the C-terminus of MIT-I (Fig. 3b–c). The linker buries 889 Å^2^ of solvent-accessible surface area as it docks within the pore. Glu97 is the last residue can be identified in the map. The remaining residues 98–108 preceding the AAA domain are not resolved, and therefore it is not possible to say to which of subunits A-F the linker is connected on its C-terminal side. The distance from Glu97 to the first ordered residue (109) of each ATPase domain ranges approximately from 19~26 Å, in each case within the maximum ~27.5 Å span of an 11-residue unstructured polypeptide. The MIT-AAA^**+**^ linker adopts an extended conformation and packs closely against VPS4B subunits A-F (Fig. 3b–c). Pore loop 1 Trp208 and Leu209 residues of each of the six subunits contacts the MIT-AAA^**+**^ linker. In addition, pore loop 2 of subunits A and F are also involved in the interaction with MIT-AAA^**+**^ linker. The side chain of subunit A Asn246 residue forms hydrogen bond with the backbone carbonyl group of MIT-I Gln91 residue, and the hydroxyl group of subunit F Ser248 residue forms two hydrogen bonds with the backbone carbonyl groups of MIT-I Ala83 and Lys81 (Fig. 3b–c). The MIT-AAA^**+**^ linker occupies a similar volume of space and interacts with the same pore loop residues as seen in the single peptide bound form of the yeast Vps4 hexamer and the transitional VPS4B hexamer described above, but the N-to-C orientation of the MIT-AAA^**+**^ linker is reversed relative to the yeast structure^32^. The N-to-C orientation of the MIT-AAA^**+**^ linker is also reversed relative to the pore loop 1-bound first strand of the N-terminal tag sequence seen in the processing state described above.

### The processing and primed states are independent of VTA1

To determine if our results were affected by the presence of VTA1, we collected a dataset using only the VPS4B^E235Q^ hexamer in the absence of VTA1. The results show that both primed and processing states were obtained at similar populations as in the VTA1-containing dataset, and also in the same conformations as in the VPS4B^E235Q^/VTA1 complex dataset (Extended Data Fig. 5). Since the structural architectures of VPS4B^E235Q^ hexamer in the absence of VTA1 are the same with VPS4B^E235Q^ hexamer in the presence of VTA1, and the resolution of VPS4B^E235Q^ hexamer in the presence of VTA1 is better, we used VPS4B^E235Q^/VTA1 complex dataset for further structural analysis in this study.

### Function of MIT-AAA^+^ linker docking in the central pore

We considered that the MIT-AAA^+^ linker sequence occupancy in the pore might have positive, negative, or even dual regulatory roles. The linker might have a positive role in promoting the initial transition from monomer or dimer to hexamer. On the other hand, its continued presence is clearly not compatible with substrate processing, and if it were to remain in the pore it would have an autoinhibitory effect. We designed linker mutations and deletions based on the interaction within the central pore (Extended Data Fig. 8). The VTA1-stimulated ATP hydrolysis rates show that two specific site-mutation V87R and K88A have a lower activity than wild-type protein, indicating that the presence of the MIT-AAA linker influences the ATP hydrolysis even far from the catalytic site (Fig. 3d). Moreover, the ΔMIT and ΔMIT+ΔLinker (ΔML) truncations also impair the enzymatic activity, implying the MIT domain could also stimulate ATP hydrolysis (Fig. 3d). To further validate the structural roles of MIT-AAA^+^ linker, we used mass photometry to measure the oligomeric state. Wild-type VPS4B mainly exists in monomer and dimer in the presence of ATPγS. However, mutations V87R and K88A cannot assemble hexamer (Fig. 3e). In the same condition, ΔMIT mainly exists in dimer, with hexamer in ~20% population, but ΔML is almost purely monomeric (Fig. 3e). These observations established that there is a positive role for the MIT and MIT-AAA^+^ linker in promoting hexamerization. They also suggest that dimerization is part of a stepwise process on the pathway to the hexamer, with the most drastic mutation, the combined MIT and linker deletion, preventing even dimerization.

### Function of theMIT-AAA^+^ interactions

**Figure 4:**
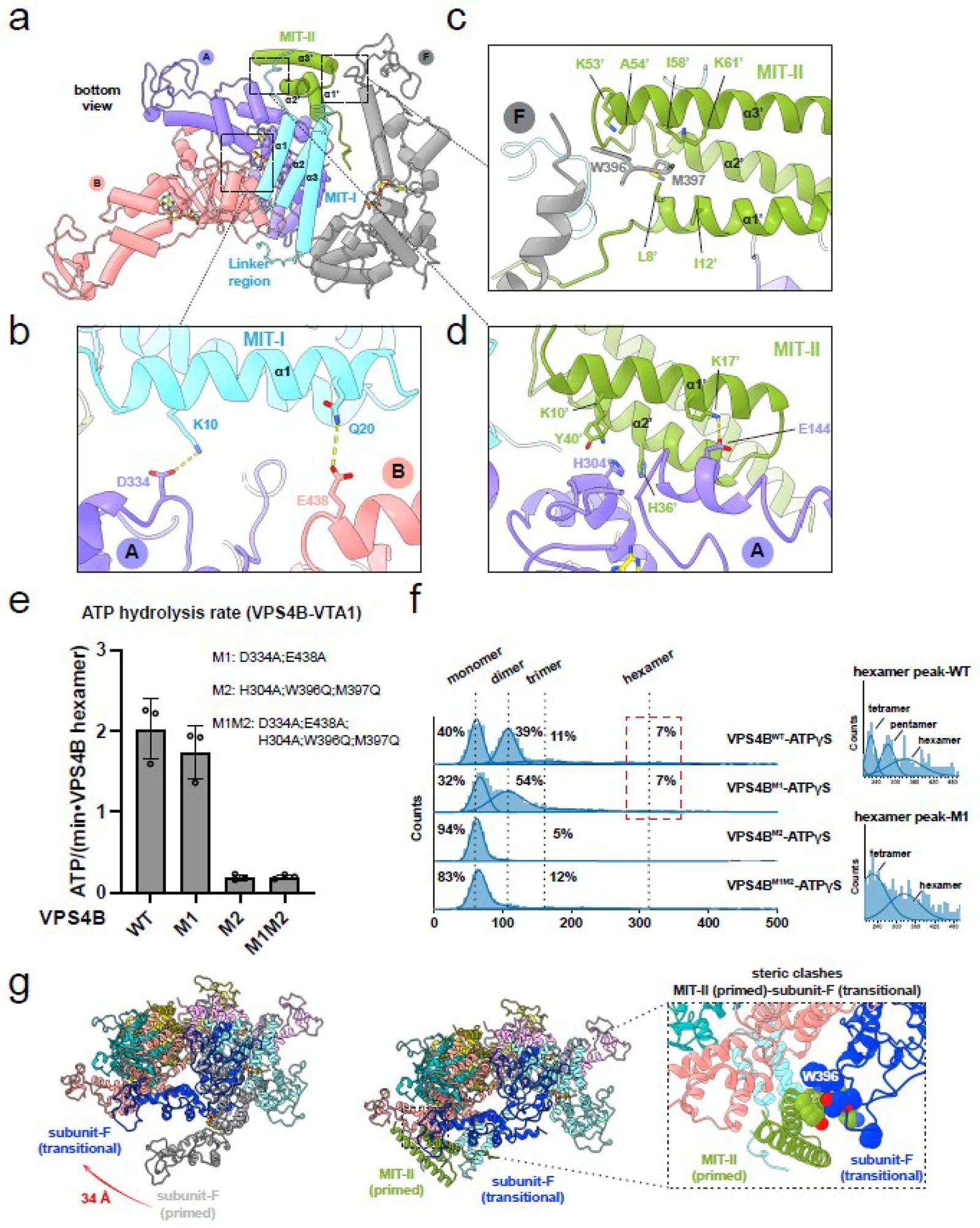
Interface between MIT domain and hexameric core. **a**, Bottom view of VPS4B primed state structure, only showing the subunits involving in the MIT docking interface. **b**, Close-up view showing the detailed interactions between MIT-I and subunits A and B in primed state structure. The related hydrogen bonds involved in the binding are shown as dotted lines. **c**, Close-up view showing the hydrophobic interactions between MIT-II and subunit F in primed state structure. **d**, Close-up view showing the detailed interactions between MIT-II and subunit A in primed state structure. **e**, ATP hydrolysis rate of human VPS4B and corresponding mutations on MIT docking interface in the presence of human VTA1. A total of 0.8 μM VTA1 was incubated with 0.4 μM VPS4B and 1 mM ATP for 30 min at room temperature (n = 3 independent experiments). **f**, Mass photometry measurements of VPS4B and related mutations incubated with 1 mM ATPγS for 30 min at room temperature in the absence of human VTA1. Inset shows the zoom-in area of hexamer in wild-type and M1 mutation protein profile. A total of 80 nM protein is used for measurements. **g**, Superposed VPS4B hexamer primed state and transitional state, showing a drastic conformational change of subunit F, which also resulting in a steric hindrance between MIT-II domain and subunit F in transitional state. Close-up view showing the detailed residues steric clashes, mainly induced by Trp396 on subunit F.

The primed state structure has the striking finding that two VPS4B MIT domain associate in an ordered manner with AAA^+^ hexamer core (Fig. 4a). There has been a long-held view that MIT domains are likely to engage with the core, on the basis of the strong contribution of MIT-ESCRT-III interactions to VPS4 regulation, yet such interactions were not observed in experimental structure determinations until now. MIT-I contacts subunits A and B simultaneously on the left-hand side of the seam as viewed in Fig. 3a. The MIT-I interface includes a salt bridge between Lys10_MIT-I_-Asp334_subunit A_, and a hydrogen bond between the side chain of MIT-I Gln20 and that of subunit B Glu438 residue (Fig. 4b). Overall, MIT-I has few direct contacts with the ATPase core and is mainly held in place by its interactions with MIT-II. MIT-II has extensive interactions with subunits A and F that span the seam of the hexamer (Fig. 4a). The side chains of subunit F Trp396 and Met397 residues of the b domain pack against the hydrophobic groove formed by the side chains of MIT-II Leu8, Ile12, Ala54, and Ile58 residues, and the aliphatic parts of the side chains of Lys53 and Lys61 (Fig. 4c). The amino group of MIT-II Lys53 residue also forms cation-π interaction with the indole ring of subunit F Trp396 (Fig. 4c). The imidazole ring of subunit A His304 forms a polar cluster with the side chains of MIT-II His36 and Tyr40 on the α2 helix, and there is a salt bridge between Lys17_MIT-II_ and Glu144_subunit A_ (Fig. 4d). The residues involved are conserved in yeast and beyond, suggestive that the MIT-AAA interaction is biologically important and conserved in evolution (Extended Data Fig. 9).

To investigate the function of the MIT domain-AAA^**+**^ interactions in the primed structure, we designed two sets of mutations to probe the MIT-I docking interface (M1: D334A; E438A; Fig. 4b) and MIT-II docking interface (M2: H304A; W396Q; M397Q; Fig. 4c, d), respectively. We also made a mutant that combined both individual sets (M1M2). We first measured the VTA1-stimulated ATPase activity of these mutants. Mutations impacting the MIT-I docking interface alone, which is, in any case, tenuous, did not have a significant effect (Fig. 4e). However, mutations impacting the MIT-II docking interface severely reduced the VPS4B enzyme activity (Fig. 4e). His304 is surface exposed in subunits B-F, and the same is true for residues 396–397 is subunits A-E. Moreover, residues 396–397 are remote from the b domain surface involved in VTA1 binding^28,55^. Therefore, these residues appear to be uniquely required for the MIT-II seam-bridging interaction, and the loss of activity is only reasonably explained by a breach of the MIT-II-AAA^**+**^ interfaces.

Mutations impacting both of the docking interfaces impair the enzyme activity to the same extent as MIT-II alone, indicating the second MIT domain is the most critical (Fig. 4e). We do not infer that MIT-I is unimportant, but rather hypothesize that MIT-I is sufficiently strongly anchored via the MIT-AAA^**+**^ linker sequence that the M1 mutation may have little or no net effect on the ability of MIT-I to dock on the ATPase core. On the basis of the role in bridging the seam, we hypothesized that the MIT-II interface is important for stabilization of the hexamer during its initial assembly. Mass photometry measurements show that the profile of M1 mutation is almost the same as wild type, whereas the M2 and M1M2 mutations drive VPS4B almost entirely into the monomeric state (Fig. 4f), similar to the combined MIT and MIT-AAA linker deletion. Therefore, the engagement of MIT-II is necessary for hexamer assembly, and presumably is responsible both directly for the engagement of MIT-II, and indirectly, for that of MIT-I with the ATPase core.

We compared the primed and transitional states to understand how the MIT domains and the MIT-AAA^**+**^ linker could be dislodged once the priming step is completed. Subunits A-E are nearly superimposable between the primed and transitional structures, but subunit F moves by up to 34 Å (Fig. 4g). The movement destroys the major MIT-II interface with subunit F Trp396 and Met397. It also leads to a steric clash between the subunit F b domain and MIT-II helix a1. ATP hydrolysis and nucleotide dissociation from subunit F should in principle be sufficient to drive the reaction cycle out of the primed state and so liberate the central pore for substrate translocation.

### MIT interface mutants disrupt VPS4B cellular function

**Figure 5:**
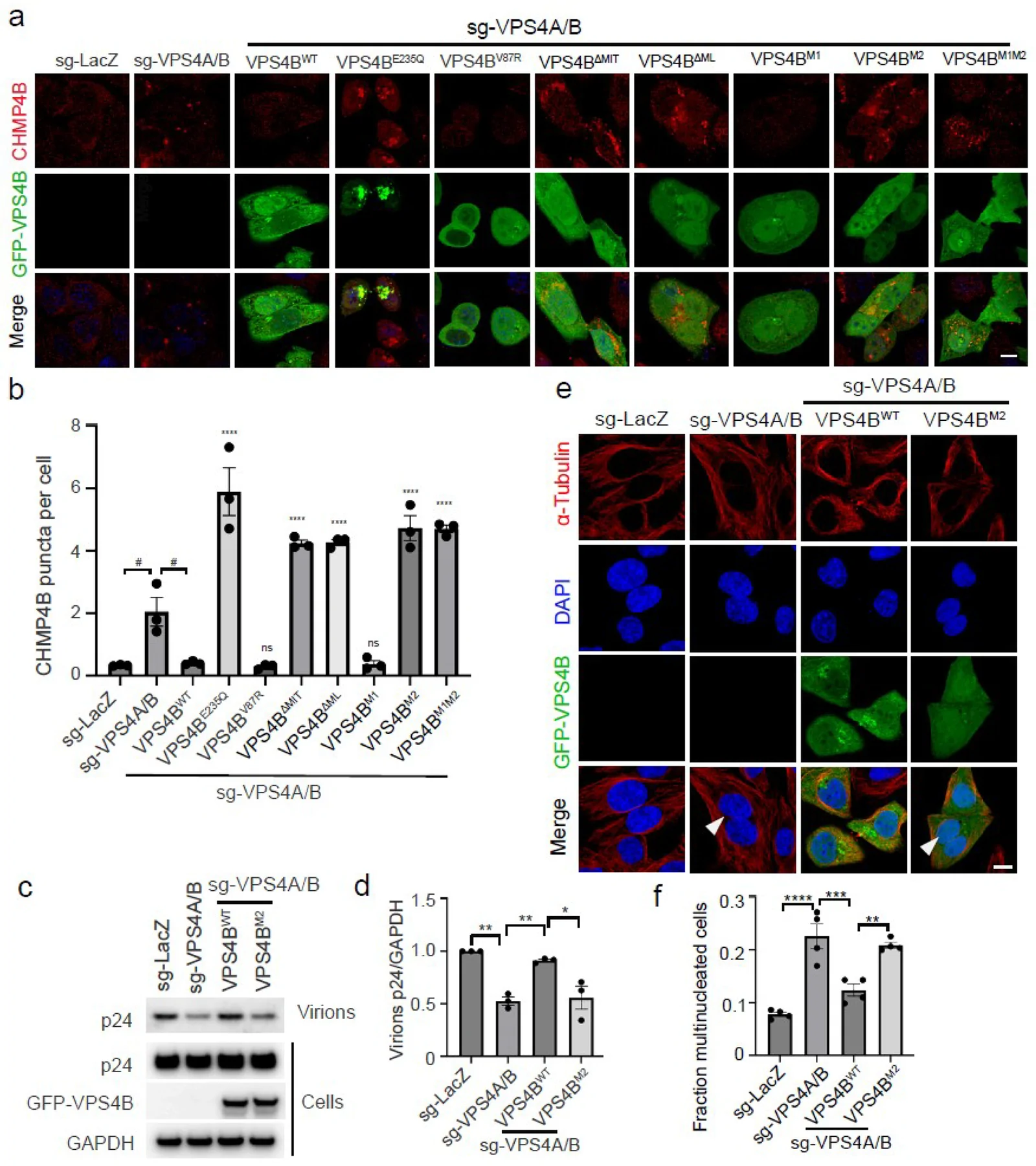
Cellular functions of VPS4B MIT interface mutants. **a**, HeLa cells were infected with sg-VPS4A/B lentiCRISPR virus to knock down endogenous VPS4A and VPS4B. Cells were then transfected with the indicated GFP-tagged VPS4B constructs (WT, E235Q, V87R, ΔMIT, ΔML, M1, M2, and M1M2) for an additional 24 h, followed by immunostaining to detect CHMP4B puncta. **b**, Quantification of the number of CHMP4B puncta per cell in (**a**) (n = 3 independent biological experiments, 160–380 cells per group were quantified). Significance between sg-LacZ and sg-VPS4A/B, and between sg-VPS4A/B and sg-VPS4A/B expressing VPS4B^WT^ is denoted as #. Significance among the various GFP-tagged VPS4B constructs under VPS4A/B knockdown condition is denoted as * (compared to VPS4B ^WT^). **c**, Lenti-X 293 cells were infected with sg-VPS4A/B lentiCRISPR virus to knock down endogenous VPS4A and VPS4B. Cells were then transfected with the iGFP-Gag and GFP-VPS4B^WT^, or GFP-VPS4B^M2^ plasmids for an additional 24 h. Western blot was performed to detect the protein level of p24 in both cell culture supernatant and cell lysate. **d**, Ratio of p24 in cell culture supernatant to GAPDH in cell lysate from (**c**) was quantified (n = 3 independent biological experiments). **e**, HeLa cells were infected with sg-VPS4A/B lentiCRISPR virus to knock down endogenous VPS4A and VPS4B. Cells were then transfected with GFP-VPS4B^WT^, or GFP-VPS4B^M2^ plasmids for an additional 24 h, followed by immunostaining with an α-Tubulin antibody to evaluate multinucleated cells (indicated by white arrows). **f**, Ratio of multinucleated cells in (**e**) was quantified (n = 4 independent biological experiments, 365–534 cells per group were quantified). Statistical significance was determined by one-way ANOVA followed by Tukey's multiple comparisons test. Scale bar:10 μm.

To investigate whether MIT interface mutants affect VPS4B function in cells, we used lentiCRISPR to knock out endogenous VPS4A and VPS4B (Extended Data Fig. 10). We then re-expressed various GFP-tagged VPS4B constructs to assess whether these mutants could rescue VPS4 cellular functions. Loss of VPS4 is known to cause aberrant accumulation of persistent CHMP4B puncta^47^, as originally observed for the yeast ortholog Snf7^24^. As expected, persistent CHMP4B puncta were detected upon VPS4A/B knockdown (Fig. 5a). Re-expression of wild-type VPS4B, V87R, and M1 mutants restored puncta disassembly, whereas the ΔMIT, ΔML, M2, and M1M2 mutants failed to disassemble CHMP4B puncta, like the E235Q dominant-negative mutant (Fig. 5a–b).

VPS4 is required for the release of HIV-1 virions from the plasma membrane of infected cells^3,51,56–60^. We performed an HIV-1 release assay, and the result showed that re-expressed VPS4B^WT^, but not VPS4B^M2^, rescued HIV-1 release in VPS4A/B knockdown cells (Fig. 5c–d). Furthermore, VPS4-mediated ESCRT-III disassembly is also required for cytokinesis, as impairment of this activity blocks the abscission step that physically separates daughter cells, consequently resulting in the formation of multinucleated cells^9,61–63^. VPS4B^WT^, but not the M2 mutant, restored a normal rate of successful cell division under VPS4A/B knockdown conditions (Fig. 5e–f). Taken together, these results confirm that the MIT interface mutants impair VPS4B activity and inhibits ESCRT function across multiple cell processes.

## Discussion

VPS4 enzymes function as hexamers. In contrast to many other AAA^+^ enzymes^23,64^, VPS4 assembles transiently in response to activating stimuli, such as the presence of polymerized ESCRT-III. The structure and biophysics of the yeast Vps4 hexamer has been intensively studied for more than a decade. Yet no such data have been available for human, or any other mammalian, VPS4 hexamer. This gap has persisted, despite the high relevance to many areas of human cell biology and disease and interest in therapeutic targeting of human VPS4 isoforms for both activation and inhibition. Moreover, despite extensive indirect evidence for MIT-AAA^**+**^ regulatory interactions, and their potential importance in biological regulation, such interactions have never been visualized for a VPS4 from any species. Here, we determined three states of human VPS4B structures and reveal previously unrecognized structural features that illuminate general aspects of VPS4 activation. Our structure of the primed state reveals the existence of a previously unappreciated step in VPS4 activation and uncovers two entirely unexpected features. First, two of the six MIT domains engage directly with the hexameric ATPase core, forming a defined docking state. Second, the MIT-AAA^**+**^ linker occupies the central pore of the hexamer in an ordered conformation. Biochemical and cell biological readouts confirm these interactions are important for VPS4 activation. These observations show that MIT domains and the MIT-AAA^**+**^ linker region synergistically promote hexamer assembly. These data expand the functional role of MIT domain beyond substrate recognition and indicate its direct involvement in stabilizing the uniquely transient hexamerization of VPS4.

In addition to the unique regulatory aspects of MIT and MIT-AAA^**+**^ linker interactions, many other aspects of the structure mirror patterns seen previously for yeast Vps4 and myriad other AAA^+^ ATPases^23,64^. The VPS4B-Hcp1 structure with its five spiral staircase subunits, and sixth transitional seam subunit, represents a most common pattern seen across all cryo-EM structures of AAA^+^ ATPases^23,64^. The nature of the subunit interfaces is highly conserved with yeast Vps4, despite that the sequences are only ~60% identical and that one structure used an EQ mutation and ATP, while the other used wild-type enzyme bound to ADP·BeF_x_. The observation of six well-ordered ATP-bound subunits in the spiral conformation engaged with a substrate-mimetic is another archetypal pattern^23,64^. The ordered double MIT docking arrangement is compatible with the six-subunit spiral in the primed structure, but not the position of the F-subunit in the seam in the more typical transitional state. The functional validation of the MIT domain interfaces in the primed structure serves also as validation of the functional relevance of the ordered six-subunit spiral. On the basis of the analogies to previously established patterns for yeast Vps4, the larger AAA^+^ family, and the validation presented here for the novel MIT domain docking mode, we believe that all three of the structures reported here represent states that are part of the VPS4 reaction cycle across species.

**Figure 6:**
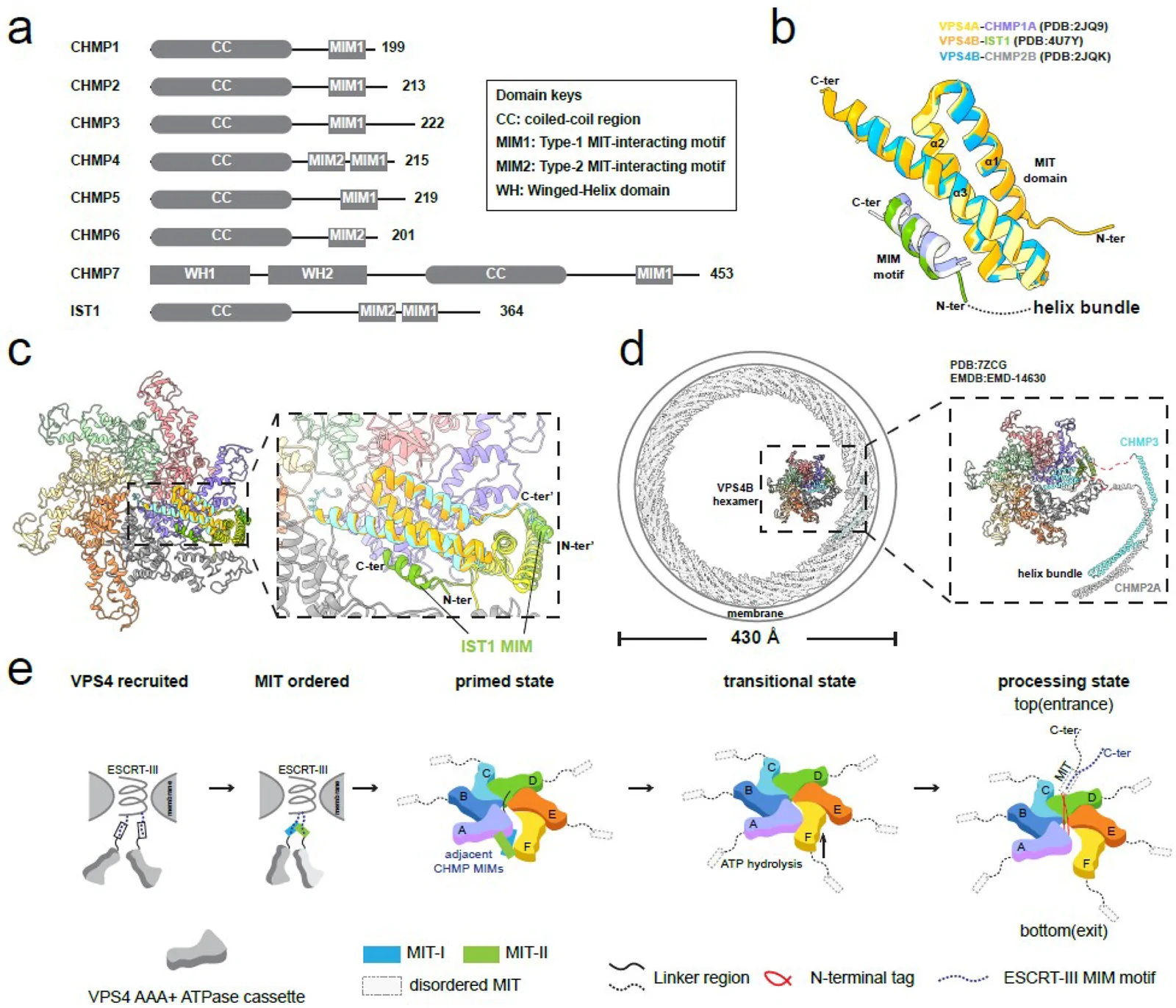
Model for VPS4B activation. **a**, Domain organization of the 12 human ESCRT-III proteins. Yeast Snf7 (the yeast version of charged body protein 4 (CHMP4)), CHMP2B and CHMP1B are depicted as representatives of the CHMP4A-CHMP4C, CHMP2A-CHMP2B and CHMP1A-CHMP1B families, respectively. The C-terminus of ESCRT-III proteins carry microtubule interaction and transport (MIT) domain-interacting motifs (MIM1 and/or MIM2), which are responsible for binding to other components of the ESCRT pathway, such as vacuolar protein sorting-associated 4 (VPS4). CHMP7 is a unique ESCRT-III protein, with its N-terminal WH domain responsible for keeping monomeric state by autoinhibited folding^10^. **b**, Structure alignment of different MIT-MIM structure complex (PDB: 2JQ9, 4U7Y, 2JQK)^51,52^, showing the same orientation of MIM motif. **c**, Structure alignment of VPS4B primed state structure with MIT-MIM structure complex. In this drawing, VPS4B^MIT^/IST1^MIM^ complex structure (PDB: 4U7Y)^52^ is aligned with MIT-I and MIT-II, showing the MIT-MIM interface is exposed to solvent in MIT docking structure. **d**, Combined EM map and structure representation showing the VPS4B hexamer located inside the ESCRT-III ring (EMDB: EMD-14630)^26^. Zoom-in panel shows the two ordered MIT domain in MIT docking structure are both accessible to interact with CHMPs MIM motif. **e**, Proposed model for human VPS4 activation.

In human cells, there are twelve ESCRT-III proteins^6,18,19^. They consist of an N-terminal helical hairpin region that can assemble on membrane necks, and C-terminal regions that contain autoregulatory sequences and VPS4-binding MIM motifs (Fig. 6a). By overlaying the primed structure on preexisting crystal structures of human VPS4 MIT-MIM complexes^51,52^, we found that the MIT-MIM1 binding grooves are exposed to for both MIT-I and -II. The gap between MIT-1 and subunit F can accommodate a MIM1 motif without steric hindrance. The C-terminus of the ESCRT-III MIM motif is positioned proximal to the bottom of the central pore (Fig. 6b–c), although the pore is blocked by the MIT-I linker sequence in the primed state. When placed in the context of the CHMP2A-CHMP3 membrane-reconstituted structure^26^, the VPS4B hexamer fits naturally on the inner side of the ESCRT-III filament. In this configuration, the C-terminal extensions of ESCRT-III subunits face inward, consistent with MIT engagement from the cytosolic side (Fig. 6d). This model aligns with the established reverse-topology scission paradigm in which VPS4 assembles on the surface distal to the membrane and contiguous with the cytosol and drives membrane constriction by extracting and threading ESCRT-III subunits through its central pore^18,19,26^.

While our study answers several previously unresolved questions in the VPS4 field, the findings also raise a new one: How does the primed state resolve into the processing state? The structure allows us to propose a model in which VPS4 monomers are recruited by CHMP MIMs following the oligomerization of ESCRT-III proteins on their membrane substrates. The close spatial proximity of two MIMs on the polymerized ESCRT-III platform promotes VPS4 dimerization. The nucleus created by the double MIT assembly then promotes hexamerization by filling the gap that would otherwise be left open across the seam of the hexamer. Insertion of the MIT-I-AAA^**+**^ linker further stabilizes the hexamer. One remaining question concerns the role of the VTA1 MIT domains, which were present but not visualized in our structure. Clearly, they have a role in avidity for the ESCRT-III substrate, but whether they have additional regulatory functions, such as the one discovered here for the VPS4 MIT domain, is still unknown. At this point, VPS4 is primed for action in the sense that the hexamer is fully assembled in a productive spiral conformation, and it is localized to its site of action. The C-termini of its substrates are recruited to the opening of the pore. However, the pore itself is blocked by the presence of the MIT-AAA^+^ linker, which needs to be displaced to reach the processing state. We propose that hydrolysis of one ATP molecule by the F subunit at the bottom of the spiral breaks helical symmetry and leads to the F-subunit moving into the transitional position. The transitional position is incompatible with the MIT docking mode in the primed state. Thus, a single ATP hydrolysis event should in principle be sufficient to eject the MIT domains and drive the transition from the primed state to the processing state (Fig. 6e).

One of the most exciting therapeutic opportunities in targeting VPS4 is the concept of synthetic lethality coupled to chromosomal codeletion of VPS4A or B with tumor suppressors^47,48^. However, the nucleotide binding sites of VPS4A and B are essentially identical, suggesting that selective ATP-site directed small molecule inhibition of A or B isoforms relative to the other may be impossible. Our structural analysis shows that a series of structural rearrangements involving less-conserved protein surfaces are required for function. In principle, an intramolecular glue could freeze in place any one of the states along the reaction pathway, with far more opportunity for selective intervention against VPS4A or B. Conversely, finding means to “lubricate” the transitions by blocking potentially inhibitory substates could be an avenue to the discovery of VPS4 activators. Alternatively, it appears that the initial hexamerization step can be rate-limiting in cells, as inferred from the phenotypes of the MIT-II-AAA^**+**^ interface mutation. Small molecules that promote hexamerization might thus in principle promote higher biological flux through the ESCRT pathway.

The ESCRT system is ubiquitous in a vast area of distinct processes in membrane biology and disease^7^. This implies that defects in a general aspect of ESCRT function, such as VPS4 priming, should manifest in defects in multiple cell processes. Here, we confirmed in VPS4A/B double knockout and rescue experiments that priming-defective VPS4B priming mutants accumulated CHMP4B foci to a similar extent as crippling ATPase activity, evidence for a loss of ESCRT-III recycling. We went on to investigate the consequences for ESCRT-mediated cytokinesis and HIV-1 release. The same patterns of loss of function were observed in these setting, confirming the physiological relevance and generality of the priming mechanism described here.

## Methods

### Cloning and protein purification.

#### Human VPS4B purification.

Full-length, human VPS4B (codon optimized) was fused to a tobacco etch virus (TEV) cleavable N-terminal 6×His tag. The C-terminal Hcp1 fusion construct was designed the same as previous paper^33^. The corresponding plasmid was transformed into *Escherichia coli* strain Rosetta (DE3) pLys and grown in Luria-Bertani (LB) medium at 37 °C. Cell growth was monitored by optical density at 600 nm and expression was induced upon reaching 0.6 at OD_600_ by adding IPTG to a final concentration of 0.2 mM. Temperature was lowered to 16 °C and cells were harvested 16 hours later. Harvest cells were pelleted and frozen at −20 °C for storage. For purification, frozen cell pellets were thawed and resuspended in lysis buffer (50 mM HEPES [pH 8.0], 500 mM NaCl, 20 mM imidazole) supplied with 1 tablet of cOmplet EDTA-free Protease Inhibitor Cocktail [Roche]. Resuspended cells were lysed by sonication, and the resulting lysate was clarified by centrifugation (45,000 × g for 30 minutes). Clarified lysate was applied to an Ni affinity column (Pierce High Capacity Ni-IMAC Resin, EDTA-compatible) that was equilibrated with lysis buffer. Bound protein was washed with lysis buffer and eluted with elution buffer (50 mM HEPES [pH 8.0], 500 mM NaCl, 300 mM imidazole). The eluate was diluted with 50 mM HEPES (pH 8.0) until reaching a final NaCl concentration of 50 mM. The diluted eluate was then applied to a HiTrap Q HP anion exchange column that was equilibrated with Anion Exchange Buffer A (50 mM HEPES [pH 8.0]). After application, the Q column was eluted with a linear gradient from 50 mM to 1 M NaCl. Elution was monitored by UV absorbance at 280 nm and fractions were assayed by SDS-PAGE. Fractions containing pure human VPS4B were pooled, concentrated, and applied to a HiLoad 16/60 Superdex 200 (Cytiva) that was equilibrated in SEC200 buffer (20 mM HEPES [pH 8.0], 100 mM NaCl, 1 mM TCEP). Elution was monitored by UV absorbance at 280 nm. Peak fractions were assayed by SDS-PAGE. Clean protein was flash frozen in liquid nitrogen and stored at −80 °C for long term use. All VPS4B variants (different mutants and truncations) were purified in an identical manner. The VPS4B constructs are as follows: VPS4B(84–444) (referred to VPS4B^ΔMIT^) and VPS4B(105–444) (referred to VPS4B^ΔML^).

#### Human VTA1 purification.

Full-length, human VTA1 was fused to a tobacco etch virus (TEV) cleavable N-terminal 6×His tag and transformed into *Escherichia coli* strain Rosetta (DE3) pLys and grown in Luria-Bertani (LB) medium at 37 °C. The following steps of protein purification is in the same way as human VPS4B.

#### VPS4B^E235Q^/VTA1 complex equipment.

To assemble VPS4B^E235Q^/VTA1 complex, VPS4B^E235Q^ and VTA1 were combined to a ~1:2 ratio and also incubate with 1 mM ATP, 2 mM MgCl_2_ for 30 min, and the assembled complex was subjected to a second round of purification on a Superdex 200 Increase 10/300 column (Cytiva) pre-equilibrated with 20 mM HEPES (pH 8.0), 100 mM NaCl, and 1 mM TCEP. Peak fractions were collected, and the protein was used immediately for cryo-EM analysis or snap frozen at −80 °C.

### Mass photometry

Protein samples were prepared in buffer with 20 mM HEPES (pH 8.0), 100 mM NaCl, 1 mM TCEP, 1 mM ATP, 2 mM MgCl_2_ and incubate 30 min at 4 °C. After incubation, a volume of 10 μL of mass photometry buffer (20 mM HEPES [pH 8.0], 100 mM NaCl, 1 mM TCEP) was added to the well for autofocus. The protein sample was then diluted in the mass photometry buffer in the wells to a concentration of 200 nM before data collection. Protein contrast counts were acquired using a Refeyn TwoMP mass photometer, with three technical replicates. Mass calibration was performed using a standard mix of conalbumin, aldolase and thyroglobulin. Mass photometry profiles were analyzed and fitted to multiple Gaussian peaks, with the mean, s.d. and percentages calculated using DiscoverMP software (Refeyn).

### Cryo-EM sample vitrification and data acquisition.

#### VPS4B^E235Q^-Hcp1 protein.

Cryo-EM samples were prepared by applying 3 μL of 1 mg/mL VPS4B^E235Q^-Hcp1 in buffer with 20 mM HEPES (pH 8.0), 100 mM NaCl, 1 mM ATP, 2 mM MgCl_2_, and 1 mM TCEP onto a grid freshly glow-discharged in PELCO easiGlow system (glow 30 s in air at 15 mA and 0.37 mbar). The holey carbon grid (C-flat, 1.2/1.3, 300 mesh) was used in this study. The samples were vitrified using an FEI Vitrobot Mark IV (Thermo Fisher Scientific) and were blotted for 3 seconds with a blot force of 10 using one Whatman 595 paper on each side of the sample at 6 °C with 95% relative humidity. The dataset was recorded using a Titan Krios G3 microscope (Thermo Fisher Scientific) equipped with a Gatan Quantum energy filter (slit width 20 eV) operated at 300 kV. Automated data acquisition was performed using SerialEM^65^ on a K3 Summit direct detection camera (Gatan) in super-resolution correlated-double sample model with a pixel size of 0.424 Å and a defocus range of −0.5 to −2.5 μm. The beam intensity was adjusted to a dose rate of approximately 1 e- per Å^2^ per frame for a 50-frame movie stack. A total of 18,567 movies were recorded.

#### VPS4B^E235Q^ hexamer determined in the presence of VTA1.

Cryo-EM samples were prepared by applying 3 μL of 0.6 mg/mL VPS4B^E235Q^/VTA1 complex in buffer containing 20 mM HEPES (pH 8.0), 100 mM NaCl, 1 mM TCEP, 1 mM ATP, and 2 mM MgCl_2_ onto a grid freshly glow-discharged in PELCO easiGlow system (glow 30 s in air at 25 mA and hold for 10 s). The holey carbon grid (Quantifoil R2/1 300-mesh [EMS]) was used in this study. The samples were vitrified using an FEI Vitrobot Mark IV (Thermo Fisher Scientific) and were blotted for 3 seconds with a blot force of 0 using one Whatman 595 paper on each side of the sample at 4 °C with 100% relative humidity. The dataset was recorded using a Titan Krios G2 microscope (Thermo Fisher Scientific) equipped with a Gatan Quantum energy filter (slit width 20 eV) operated at 300 kV. Automated data acquisition was performed using SerialEM^65^ on a K3 Summit direct detection camera (Gatan) in super-resolution correlated-double sample model with a pixel size of 0.472 Å and a defocus range of −0.8 to −2.5 μm. The beam intensity was adjusted to a dose rate of approximately 1 e- per Å^2^ per frame for a 50-frame movie stack. A total of 13,699 movies were recorded.

#### VPS4B^E235Q^ hexamer determined in the absence of VTA1.

We collected a dataset for the VPS4B^E235Q^ hexamer in the absence of VTA1, and the results show that both primed and processing states could be obtained as well, also exhibiting no difference with VPS4B^E235Q^-VTA1 complex dataset other than maximum resolution attained (Extended Data Fig. 5). Since the resolution of VPS4B^E235Q^ hexamer in the presence of VTA1 was higher, and no other differences were noted, we used the VPS4B^E235Q^-VTA1 complex dataset in this study. Cryo-EM samples were prepared by pre-incubating VPS4B^E235Q^ with 1 mM ATP, 2 mM MgCl_2_ for 30 min, and then applied 3 μL of 0.6 mg/mL VPS4B^E235Q^ onto a grid freshly glow-discharged in PELCO easiGlow system (glow 30 s in air at 25 mA and hold for 10 s). The holey carbon grid (Quantifoil R1.2/1.3 300-mesh [EMS]) was used in this study. The samples were vitrified using an FEI Vitrobot Mark IV (Thermo Fisher Scientific) and were blotted for 3 seconds with a blot force of 0 using one Whatman 595 paper on each side of the sample at 4 °C with 100% relative humidity. The dataset was recorded using a Titan Krios G2 microscope (Thermo Fisher Scientific) equipped with a Gatan Quantum energy filter (slit width 20 eV) operated at 300 kV. Automated data acquisition was performed using SerialEM^65^ on a K3 Summit direct detection camera (Gatan) in super-resolution correlated-double sample model with a pixel size of 0.472 Å and a defocus range of −0.8 to −2.5 μm. The beam intensity was adjusted to a dose rate of approximately 1 e- per Å^2^ per frame for a 50-frame movie stack. A total of 5,360 movies were recorded.

### Cryo-EM processing and 3D reconstruction

#### VPS4B^E235Q^-Hcp1 hexamer (transitional state).

The data processing workflow for VPS4B^E235Q^-Hcp1 fusion constructusing cryoSPARC(v.4)^66^ is shown in Extended Data Fig. 1, and statistics is summarized in Table 1. In summary, Patch motion correction was used for motion correction and Patch contrast transfer function (CTF) estimated was used for CTF determination. Particles were automatically picked by Topaz^67^ using a manually trained model and extracted with a window size 1.5 times larger than the target particle before further binning 2× to 4× to facilitate the following processing. The resulting particles underwent iterative 2D-classification to remove obvious ‘junk’ and all classes exhibiting protein-like features (2,793,289 particles) were retained. The Hcp1 fusion construct has a preferential orientation issue and was biasedly picked the side view of particles resulting in a subset of 430,866 particles. Particles were used to generate four reference maps by ab initio, following with iterative heterogeneous refinement to a subset of 127,996 particles and a round of homogeneous refinement resulted in a 2.96 Å map at 0.143 Fourier shell correlation (FSC). Mask was generated surrounding the Hcp1 part using UCSF ChimeraX and imported into cryoSPARC and was used for the following particle subtraction. The resulting subtracted particles underwent another round of 2D-classification to remove obvious ‘junk’ and all classes exhibiting protein-like features (118,899 particles) were retained. Particles were used to generate three reference maps by ab initio, following with iterative heterogeneous refinement to final subset of 66,315 particles and a round of non-uniform refinement resulted in a 2.90 Å map at 0.143 Fourier shell correlation (FSC).

#### VPS4B^E235Q^ hexamer (VPS4B^E235Q^/VTA1 complex).

The data processing workflow for VPS4B “primed” and “processing” state structures using cryoSPARC(v.4)^66^ are shown in Extended Data Fig. 4d, and statistics are summarized in Table 1. In summary, Patch motion correction was used for motion correction and Patch contrast transfer function (CTF) estimated was used for CTF determination. The blob picker with a diameter range of 130–256 Å was used to pick 7,325,906 particles, which were trimmed to 5,795,804 particles by Inspect picks. Particles were extracted with a box size of 360 pixels further binning 4× to 90 pixels to facilitate the following processing. The resulting particles underwent iterative 2D-classification to remove obvious ‘junk’ and all classes exhibiting protein-like features (2,557,837 particles) were retained. Particles were used to generate four reference maps by ab initio, following with iterative heterogeneous refinement to a subset of 103,596 particles and a subset of 882,583 particles for further processing.

The less populated subset particles were re-extracted using a box size of 256 pixels and a round of non-uniform refinement^68^. Then, the resulting map was applied to a round of 3D-classification to further clean the subset, resulting in the final subset containing 20,535 particles, following with global and local CTF refinement^69^, reference based motion correction^70^, and final round of non-uniform refinement resulting in a 3.32 Å map (primed) at 0.143 Fourier shell correlation (FSC).

The more populated subset particles were re-extracted using a box size of 256 pixels and a round of non-uniform refinement^68^, following with global and local CTF refinement^69^, reference based motion correction^70^, and a round of homogenous refinement. Mask was generated focusing on the central pore using UCSF ChimeraX and imported into cryoSPARC with two rounds focused 3D-classification, and the final round of non-uniform refinement resulting in a 2.69 Å map (processing) at 0.143 Fourier shell correlation (FSC).

#### VPS4B^E235Q^ hexamer (without VTA1).

The data processing workflow for VPS4B “primed” and “processing” state structures using cryoSPARC(v.4)^66^ are shown in Extended Data Fig. 5, and statistics are summarized in Table 1. In summary, Patch motion correction was used for motion correction and Patch contrast transfer function (CTF) estimated was used for CTF determination. Particles were automatically picked by Topaz^67^ using a manually trained model and extracted with a window size 1.5 times larger than the target particle before further binning 2× to 4× to facilitate the following processing. The resulting particles underwent iterative 2D-classification to remove obvious ‘junk’ and all classes exhibiting protein-like features (416,446 particles) were retained. Particles were used to generate four reference maps by ab initio, following with iterative heterogeneous refinement to a subset of 173,071 particles for further processing. Then, mask was generated surrounding the hexamer seam using UCSF ChimeraX and imported into cryoSPARC and was used for the following focused 3D-classification, resulting in two particle subsets.

The less populated subset particles were re-extracted using a box size of 256 pixels and a round of 3D-classification to further clean the subset, resulting in the final subset containing 12,231 particles, following with global and local CTF refinement^69^, reference based motion correction^70^, and final round of non-uniform refinement^68^ resulting in a 3.76 Å map (primed) at 0.143 Fourier shell correlation (FSC).

The more populated subset particles were re-extracted using a box size of 256 pixels and a round of 3D-classification to further clean the subset, resulting in the final subset containing 12,231 particles, following with global and local CTF refinement^69^, reference based motion correction^70^, and final round of non-uniform refinement^68^ resulting in a 3.11 Å map (processing) at 0.143 Fourier shell correlation (FSC).

### Atomic model building and refinement

The structural model of hexameric VPS4B^E235Q^ model was generated by AlphaFold3^71^ using six copies of VPS4B^E235Q^ and six copies of ATP. Generally, we fit this model into the final round cryo-EM map as a rigid body using UCSF ChimeraX^72^. The resulting model was further refined using Phenix real-space refinement^73,74^ with iterative inspections and adjustments using Coot^75^. Final model statistics are contained within Table 1. All figures were created using UCSF ChimeraX. For VPS4B hexamer primed state structure, two MIT domain were fitted into cryo-EM map as a rigid body using UCSF ChimeraX, and the MIT-AAA linker was manually built into the density using Coot. For the processing state structure, the N-terminal tag was manually built into the density using Coot. For VPS4B^E235Q^-Hcp1 hexamer structure (transitional state), subunits A-E were refined using Phenix real-space refinement with iterative inspections and adjustments using Coot, and subunit F was fitted into the cryo-EM map as a rigid body without further refinement because the density quality is not sufficient for side chain building. We can also observe extra density in the central pore. Given the insufficient side-chain density for sequence assignment, we modeled this region as a polyA peptide. Final model statistics are contained within Table 1.

### Structural consequences of Hcp1 fusion

We used two strategies to stabilize the VPS4B hexamer, which resulted in two related but distinct helical arrangements. In the presence of Hcp1, five subunits form a regular helix but the sixth occupies a transitional and poorly ordered position between the start and end of the helical unit (Extended Data Fig. 6a), while an intact six-unit helix is seen in both the processing and primed states for the unfused construct (Extended Data Fig. 6b-c). When we docked the unfused primed state structure onto the VPS4B^E235Q^-Hcp1 map, there is a steric clash between MIT-I and the Hcp1 density. However, upon rotating the hexamer core by one subunit, two MITs can be accommodated on the hexamer without any steric hindrance with the Hcp1 density (Extended Data Fig. 6d). By comparing two different states of VPS4B hexamer core in spiral shaped conformation, we found that they share the same architecture, and the root mean square deviation (r.m.s.d.) value of two structures is ~1.1 Å, indicating they are very similar (Extended Data Fig. 6e). The subunits A and F in primed state structure are close to each other, while the seam between subunits A and F is more open in processing state, and we think the association between MIT-II and hexamer core leads to a compact conformation in primed state structure (Extended Data Fig. 6b-c). Further comparison of each subunit shows that the overall folding resembles for both Hcp1 subtracted hexamer and Hcp1-less hexamer, but the β domain of subunit E and F undergo an ~11 Å and ~34 Å shift from the bottom face to top face, respectively, which is consistent with Hcp1 hexamer location, suggesting Hcp1 may force subunit F to be closer to bottom side (Extended Data Fig. 6f).

### Promega ADP-Glo assay

The enzyme activity was measured by the generation of ADP using Promega’s ADP-Glo^™^ Kinase Assay. Enzymes were assayed at 0.4 μM (wild type and mutants) in buffer with 50 mM HEPES (pH 8.0), 100 mM NaCl, 1 mM ATP, 10 mM MgCl_2_ with 0.8 μM full-length VTA1. Reactions were started by adding a 1.6 μM VTA1 and 2 mM ATP solution to 0.8 μM VPS4B solution. Reactions were allowed to proceed at room temperature for 30 minutes before being quenched by the addition of Promega ADP-Glo^™^ Reagent at a 1:1 ratio. Assay conditions follow the recommended timing and ratios found in the Promega ADP-Glo^™^ Kinase Assay manual. The reaction + ADP-Glo^™^ Reagent solution was incubated for 40 minutes before the addition of Promega Kinase Detection Reagent at a 1:1 ratio (reaction + ADP-Glo^™^ Reagent: Kinase Detection Reagent) and incubated for 30 minutes prior to measuring reaction luminescence.

### Cell culture

HeLa cells and Lenti-X 293 cells (obtained from UCB Cell Culture Facility) were cultured in DMEM medium supplemented with 10% FBS. The culture environment was maintained at 37 °C with 5% CO_2_ in a humidified incubator.

### Lentivirus production

Lentiviral particles were packaged in Lenti-X 293 cells using a Mirus LT1 (Mirus Bio, MIR2300)-mediated transfection protocol at 90% confluency. The transfection mixtures consisted of 6 μg sgRNA plasmids, 4.5 μg psPAX2 (Addgene, 12260), and 3 μg VSVG (Addgene, 8454). After a 48-hour incubation, the viral-containing media were collected and filtered (using a 0.45 μm syringe filter; Cytiva, 76479-020), the viral particles were concentrated using Lenti-X Concentrator (Takara Bio, 631231) to obtain high-titer stocks. The following sgRNAs were cloned into lentiCRISPR V2 plasmid (Addgene, 52961) to knock down VPS4A/B: sg-VPS4A^#1^ 5’- GTGACTCACACTTGATAGCG-3’, sg-VPS4A^#2^ 5’- AACCCTCCAGGTACTTCGTG-3’, sg-VPS4B^#2^ 5’- CCGCAACATATCCGACTGTC-3’, sg-VPS4B^#3^ 5’- TTCTGCCATTAGGCGAAGGT-3’.

### Fluorescent staining

LentiCRISPR virus was used to knock down VPS4A/B in HeLa cells as described above. The cells were subsequently replated into 8-chamber confocal dishes. The following day, Lipofectamine 3000 transfection reagent were used to transfect cells with GFP-VPS4B^WT^, GFP-VPS4B^E235Q^, GFP-VPS4B^V87R^, GFP-VPS4B^ΔMIT^, GFP-VPS4B^ΔML^, GFP-VPS4B^M1^, GFP-VPS4B^M2^, or GFP-VPS4B^M1M2^ plasmids. After 24 h, cells were washed twice with PBS and fixed with 4% paraformaldehyde (PFA) (Electron Microscopy Science, 15710) for 10 min at room temperature, followed by permeabilization with 0.1% PBST (PBS containing 0.1% Triton X-100) for 10 min. After blocking with 4% BSA (in 0.05% saponin) for 1 h, cells were incubated with primary antibodies (diluted in blocking buffer) overnight at 4 °C. Following three washes with 0.05% saponin, cells were incubated with secondary antibodies for 1 h at room temperature. After three additional washes with 0.05% saponin, cell nuclei were stained with DAPI. The cells were kept in PBS and imaged using a Nikon confocal microscope (A1R) with a 60× objective. The following primary antibodies were used: CHMP4B (Proteintech, 13683-1-AP), and α-Tubulin (Abcam, ab7291). GFP-tagged VPS4B constructs were modified from pLNCX2-mEGFP-VPS4A (Addgene, 116924)^60^, through Gibson assembly to substitute *VPS4A* as *VPS4B* gene.

### Western blot

HeLa or Lenti-X 293 cells were lysed in a 4% SDS-based buffer containing 50 mM Tris-HCl, 100 mM DTT, 12.5% glycerol, and protease inhibitors (Thermo Fisher Scientific, A32965). Lysates were heated at 95°C for 15 min and then separated by SDS-PAGE (NuPAGE 4–12% Bis-Tris gels; Invitrogen, NP0323BOX). Then proteins were transferred to membranes and probed with primary antibodies against VPS4A (Santa Cruz Biotechnology, sc-393428), VPS4B (Abcam, ab137027), HIV-1 p24 (Abcam, ab32352), and GAPDH (Proteintech, 60004-1-Ig). Signals were generated using SuperSignal West Atto substrate (Thermo Fisher Scientific, A38556) and visualized on a ChemiDoc MP imaging system (Bio-Rad).

### HIV-1 release experiment

The HIV-1 release protocol was modified from a previously study^76^. Lenti-X 293 cells were plated in 6-well dishes and allowed to adhere overnight. To silence endogenous VPS4A/B, cells were infected with sg-VPS4A/B lentiCRISPR viruses. The following day, cells were replated into 24-well plates and transiently transfected with iGFP-Gag and GFP-VPS4B^WT^, or GFP-VPS4B^M2^ plasmids using Lipofectamine 3000 transfection reagent (Thermo Fisher Scientific, 100022050) according to the manufacturer’s protocol. After 24 h transfection, cell supernatant was clarified by passage through a 0.45 μm syringe filter, then mixed with 1/4 Volume of 4× LDS sample buffer (Invitrogen, NP0008), and the cells were lysed in a 4% SDS-based buffer. The cell supernatant and cell lysate samples were denatured by heating at 95 °C for 15 min. Then western blot was performed to detect the protein level of P24 in both cell culture supernatant and cell lysate.

### Data analysis

GraphPad Prism 10 was utilized for all statistical evaluations, with data reported as means ± SEM. Statistical significance was determined by one-way ANOVA followed by Tukey's multiple comparisons test. Results reached statistical significance if *P* < 0.05. Significance levels were categorized as *P < 0.05, **P < 0.01, ***P < 0.001, and ****P < 0.0001. Non-significant results are marked as “ns”.

## Supplementary Material

Supplementary Files

This is a list of supplementary files associated with this preprint. Click to download.

- Uncroppedwesternblotsimages.pdf
- Ext1.ai
- Ext2.ai
- Ext3.ai
- Ext4.ai
- Ext5.ai
- Ext6.ai
- Ext7.ai
- Ext8.ai
- Ext9.ai
- Ext10.ai
- ExtendedDataFigureLegends.docx

## Figures and Tables

**Table 1.** Cryo-EM data collection, refinement and validation statistics

	VPS4B hexamer in primed state (EMD-75716) (PDB 11IK)	VPS4B hexamer in processing state (EMD-75713) (PDB 11IG)	VPS4B hexamer in transitional state (EMD-75714) (PDB 11II)	VPS4B hexamer in primed state (without VTA1)	VPS4B hexamer in processing state (without VTA1)
**Data collection and processing**					
Magnification	81,000		105,000	81,000	
Voltage (kV)	300		300	300	
Electron exposure (e^−^ per Å^2^)	50		50	50	
Defocus range (μm)	−0.8 to −2.5		−0.5 to −2.5	−0.8 to −2.5	
Pixel size (Å)	0.4716 (super-resolution)		0.424 (super-resolution)	0.4716 (super-resolution)	
Symmetry imposed	None		None	None	
Initial particle images (no.)	5,795,804 (blob picking)		2,961,654 (blob picking)	1,868,336 (blob picking)	
Final particle images (no.)	20,535	202,946	66,315	11,168	101,337
Map resolution (Å)	3.32	2.69	2.90	3.76	3.11
FSC threshold	0.143	0.143	0.143	0.143	0.143
Map resolution range (Å)	~2.9 to ~54.8	~2.4 to ~43.3	~2.6 to ~48.4	~3.4 to ~60.0	~2.7 to ~49.5
**Refinement**					
Initial model used	AlphaFold	AlphaFold	AlphaFold		
Model resolution (Å)	3.3	2.7	2.9		
Map sharpening B factor (Å^2^)	89.1	107.2	81.7		
Model composition					
Non-hydrogen atoms	17,410	16,034	15,651		
Protein residues	2,204	2,029	1,992		
Ligands	12	12	5		
B factors (Å^2^)					
Protein	99.99	56.69	103.02		
Ligand	61.59	23.51	57.05		
R.m.s.d.					
Bond lengths (Å)	0.002	0.002	0.003		
Bond angles (°)	0.531	0.545	0.571		
**Validation**					
MolProbity score	1.52	1.32	1.34		
Clash score	3.66	3.33	2.38		
Poor rotamers (%)	0.00	0.00	0.00		
Ramachandran plot					
Favored (%)	94.70	96.77	95.33		
Allowed (%)	5.03	3.03	4.62		
Disallowed (%)	0.27	0.20	0.05		

## Data Availability

The cryo-EM maps were deposited to the EM Data Bank (EMDB) under accession codes EMD-75716 (primed state), EMD-75713 (processing state), EMD-75714 (transitional state). The structural coordinates were deposited to the PDB under accession codes 11IK (primed state), 11IG (processing state), 11II (transitional state). All other data of this study are available from the corresponding authors on reasonable request. Source data are provided with this paper.
